# Epigenetic reactivation of LINE‐1 retrotransposon disrupts NuRD corepressor functions and induces oncogenic transformation in human bronchial epithelial cells

**DOI:** 10.1002/1878-0261.12329

**Published:** 2018-06-21

**Authors:** Pasano Bojang, Kenneth S. Ramos

**Affiliations:** ^1^ Division of Pulmonary, Allergy, Critical Care and Sleep Medicine University of Arizona College of Medicine Tucson AZ USA; ^2^ Center for Applied Genetics and Genomic Medicine University of Arizona Health Sciences Tucson AZ USA

**Keywords:** benzo(a)pyrene, euchromatin, heterochromatin, long interspersed nuclear element‐1, nucleosomal and remodeling deacetylase complex, retrotransposon

## Abstract

Long interspersed nuclear element‐1 (LINE‐1 or *L1*) reactivation is linked to poor prognosis in non‐small‐cell lung carcinoma (NSCLC), but the molecular bases of this response remain largely unknown. In this report, we show that challenge of human bronchial epithelial cells (HBECs) with the lung carcinogen, benzo(a)pyrene (BaP), shifted the *L1* promoter from a heterochromatic to euchromatic state through disassembly of the nucleosomal and remodeling deacetylase (NuRD) complex. Carcinogen challenge was also associated with partial displacement of constituent proteins from the nuclear to the cytoplasmic compartment. Disruption of NuRD corepression by genetic ablation or carcinogen treatment correlated with accumulation of *L1* mRNA and proteins. Mi2β bound directly to the *L1* promoter to effect retroelement silencing, and this response required the DNA‐ and ATPase‐binding domains of Mi2β. Sustained expression of *L1* in HBECs was tumorigenic in a human–SCID mouse xenograft model, giving rise to tumors that regressed over time. Together, these results show that functional modulation of the NuRD constituent proteins is a critical molecular event in the activation of *L1* retrotransposon. *L1* expression creates a microenvironment in HBECs that is conducive to neoplasia and malignant transformation.

**Abbreviations**
BaP: benzo(a)pyrene
HBECs: human bronchial epithelial cells
Line‐1/L1: long interspersed nuclear element‐1 (LINE‐1 or *L1*)
MBD: methyl‐CpG‐binding domain proteins
MTA: metastasis‐associated proteins
NuRD: nucleosomal and remodeling deacetylase
RNPS: ribonucleoprotein particles (RNPs)

## Introduction

Human *L1* is ~6 kb and consists of an internal promoter located within the 5′untranslated region (5′UTR), two open reading frames (ORFs) encoding ORF1p and ORF2p, and a 3′UTR with a poly(A) tail and signal (Dombroski *et al*., 1991; Scott *et al*., 1987). *L1* elements constitute a large family of mammalian retrotransposons that have continuously replicated for over 100 Myr (Boissinot *et al*., 2000). Approximately 100 retrotransposition‐competent *L1*s remain in the human genome that function as autonomous elements through a copy‐and‐paste mechanism (Brouha *et al*., 2003). The human *L1* promoter is highly conserved among active *L1*s, except for a single nucleotide deletion at position 74, and a single nucleotide polymorphism (SNP) at 711 (t/c), both of which differentiate Ta‐1d from the Ta‐Ind, Ta‐0, and Pre‐Ta families (Boissinot *et al*., 2000; Swergold, 1990). The active human Ta family arose ~4MYA and subsequently differentiated into two major subfamilies, Ta‐0 and Ta‐1, each of which contains additional members. Ta‐1 is younger than Ta‐0 and now accounts for at least 50% of the Ta family (Boissinot *et al*., 2000).

A cycle of retrotransposition involves transcription of both ORFs from a common 5′UTR by RNA *Pol*II, followed by translation in the cytoplasm (Swergold, 1990). The *L1*‐encoded proteins exhibit either *cis*‐ (major) or *trans‐* (minor) preference to bind *L1* mRNA or SINEs and other cellular mRNAs, to form ribonucleoprotein particles (RNPs) (Hohjoh and Singer, 1996; Wei *et al*., 2001). The RNP‐containing *L1* mRNA, or other mRNAs, mainly localizes to the cytoplasm, but can translocate into the nucleus where the endonuclease domain of ORF2p nicks a single strand of genomic DNA to expose a 3′‐OH group that can then be used by the reverse transcriptase domain of ORF2p to prime and reverse synthesize *L1* cDNA from the 3′ end (Feng *et al*., 1996; Hattori *et al*., 1986). This process can lead to full‐length or truncated insertions of *L1* sequences throughout the genome (Moran *et al*., 1996).

Because uncontrolled retrotransposition can be genotoxic and detrimental to the host, active *L1* elements are silenced in most somatic cells via DNA methylation and histone covalent modifications (Montoya‐Durango *et al*., 2009; Teneng *et al*., 2011). In addition, mammalian cells evolved several mechanisms against retrotransposition, including the deamination by APOBEC proteins, *L1* mRNA degradation, and preferential targeting of *L1* sequences to gene‐poor regions (Bojang *et al*., 2014; Esnault *et al*., 2006). *L1* is reactivated by tobacco carcinogens such as benzo(a)pyrene (BaP) through a process that is orchestrated by the aryl hydrocarbon receptor (AHR) and retinoblastoma (RB) family of proteins to influence the epigenetic machinery (Lu and Ramos, 2003; Montoya‐Durango *et al*., 2009; Stribinskis and Ramos, 2006; Teneng *et al*., 2007). *L1* reactivation can reshuffle the genome to afford a survival advantage or mediate acquisition of stem cell‐like properties and epithelial‐to‐mesenchymal transition (EMT), as seen in non‐small‐cell lung cancers (NSCLCs) (Bojang *et al*., 2013; Ikeda *et al*., 2013; McClintock, 1984; Saito *et al*., 2010). We recently showed that *L1* couples EMT with oncogenesis in human bronchial epithelial cells (HBECs) through a signaling cascade initiated by TGF‐β1 (Reyes‐Reyes *et al*., 2017).

Given the possible roles of *L1* in lung pathogenesis, elucidation of the molecular mechanisms of *L1* regulation is of interest. In this context, we have shown that the nucleosomal and remodeling deacetylase (NuRD) multiprotein complex is enriched in the 5′untranslated DNA sequence of *L1* in human and murine cells. Further, genetic ablation of RB proteins destabilizes interactions within the NuRD complex, and mediates nuclear rearrangement of Mi2β, an ATP‐dependent helicase subunit with nucleosome remodeling activity (Montoya‐Durango *et al*., 2016). The NuRD multiprotein complex consists of six core components, including the chromodomain‐helicase‐DNA‐binding proteins 3 or 4 (also known as *Mi2*α and *Mi2*β, respectively); methyl‐CpG‐binding domain proteins 1/2/3 (*MBD1/2/3*); metastasis‐associated proteins 1/2/3 (*MTA1/2/3*); histone‐binding proteins (Rbbp4/7, RbAp46/48), histone deacetylases 1/2 (HDAC1/2), and nuclear zinc finger proteins (*Gata2a/2b(p66*α*/*β)) (Xue *et al*., 1998; Zhang *et al*., 1999). *HDAC1/2* and *Rbbp4/7* are not exclusive to the NuRD complex and are also present in the SIN3 and CoREST multiprotein complexes, while *Mi2*α*/*β, *MBD*s, and *MTA*s are relatively specific to the NuRD complex, and their interactions with core components and other accessory proteins mediate recruitment and functionally of the NuRD complex (Humphrey *et al*., 2001).

The present studies were conducted to elucidate structural and functional determinants of NuRD corepression within the *L1* promoter in HBECs following reactivation by the tobacco carcinogen, BaP. Evidence is presented here that *L1* reactivation in HBECs by BaP shifts the *L1* promoter from a heterochromatic to euchromatic state through disassembly and delocalization of the NuRD multiprotein complex. This response leads to cellular accumulation of ORF1 and ORF2 mRNA and proteins. The corepressor function of NuRD is dependent upon the DNA and ATPase domains of *Mi2*β*,* working together with *MBD2/3* to regulate the state of *L1* chromatin. Expression of a constitutively active *L1* in HBECs was tumorigenic in a SCID mouse xenograft model, a finding that directly implicates *L1* reactivation in neoplasia and malignant transformation of lung epithelial cells.

## Materials and methods

### Plasmids and cloning

Mi2β wild‐type (WT) and a biologically inactive W508A chromodomain mutant were kind gifts from J. Hagman at the University of Colorado (Ramírez *et al*., 2012). DNA (E874A)‐ and ATPase (W879A)‐binding domain mutants were created as described previously using PCR primers as specified in Table 1 (Ramírez *et al*., 2012). Mutant fragments were joined by nested PCR and cloned into a Age I and pSHA I linearized MSCV vector by infusion cloning (Clontech). *L1*‐5′UTR promoter was cloned into the *Bgl*II site of pGL4.15 (luc2P/Hygro) vector using infusion cloning and primers listed in Table 1. Cloned fragments were sequenced in reverse and forward directions to confirm identity and orientation. HBECs were transfected with pB001^CTR^ or pB015^WT^ using Lipofectamine, as previously described. The pB015^WT^ and pB001^CTR^ (i.e., pGL4.15 backbone) vectors were used to create stably transfected HBECs constitutively expressing *L1*‐ORF1 and ORF2 proteins, as confirmed by indirect immunofluorescence.

**Table 1:** Cloning of Mi2β and L1‐5′UTR

Sequence name	Sequence
DNA (E874A)‐IF	5′‐GAAATTCCATGTGCTGCTGACAGCCTATGAATTGATC‐3′
DNA (E874A)‐IR	5′‐CATGTCAATG GTGATCAATTCATAGGCTGTCAGCAG‐3′
ATPase (W879A)‐1F	5′‐CTGAGCCCTATGCAGAAGAA ATACTACGCGTACATCCTC‐3′
ATPase (W879A)‐1R	5′‐CTTCAAAATTTCGAGTGAGGATGTACGCGTA GTATTTCTT C‐3′
	5′‐GTACAGACAGCAGTCTTCCTGTATTCCCTT TACAAGG‐3′
	5′‐GGTCACAAACCGG TAGATCATTACCTTTTTATTTTGC‐3′
pGL4.15Luc‐1F	5′‐GAGGATATCAAGATCTGGGGGGAGGAGCCAAGATGGCCG‐3′
pGL4.15Luc‐1R	5′‐GCCGAGGCCAGATCTCTTTGTGTTTTATCTACTTTT GGTC‐3′

### Real‐Time (RT) PCR, siRNA treatment, western analysis, and indirect immunofluorescence

BEAS‐2B cells (CRL#: 9609, ATCC) is a nontransformed HBEC line immortalized with a replication‐defective SV‐40/adenovirus 12 hybrid (Cat#: 15338100, Thermo Scientific). Despite the presence of T‐antigen in replication‐defective lines (Butel and Rapp, 1966), BEAS‐2B cells remain RB/p53 competent (Pan *et al*., 2011), and are representative of squamous epithelial cells within the proximal airway. The integrity of RB and p53 signaling was confirmed in studies showing intact RB phosphorylation and p53 acetylation in BEAS‐2B cells (data not shown). Cells were grown to 70% confluence and treated with scrambled siRNA or siRNAs directed against Mi2β, MBD2/3, and MTA1/2/3 for 48 h. Total RNA was extracted and 0.5–1 μg used for cDNA synthesis (Invitrogen Superscript II) with OligoDT primers (Tables 2 and 3). The homogeneity of PCR products was confirmed using an RT‐PCR melting curve for SYBR probes. For TaqMan probes, ΔΔCT was used to calculate the relative quantity of *L1*. For SYBR probes, a standard curve method was used to calculate relative quantity. For western blot analysis, HBECs were lysed with RIPA buffer and 15–30 μg total protein loaded onto 4–12% gradient gels (Invitrogen). A polyclonal antibody against *L1*‐ORF1 generated by New England Peptide and validated in previous studies was used in all experiments (Ramos *et al*., 2011; Reyes‐Reyes *et al*., 2016). The optimization of *L1*‐ORF1 detection by Western analysis in HBECs is presented in Fig. S1. The overexpression of *L1* proteins in cells transfected with pB015WT was confirmed by Western and documented previously (Reyes‐Reyes *et al*., 2017). Mi2β (Cat#: SAB5300324), MBD2/3 (Cat #: SAB2101140/SAB1300141), and MTA2/3 (Cat#: M7569, WH0057504MI) antibodies were obtained from Sigma or Cell Signaling. Primary antibodies against NuRD proteins were diluted 1/1000, while the ORF1 antibody was diluted 1/500 and incubated overnight. The antibody against GAPDH was obtained from Santa‐Cruz (Cat#: sc‐47724). Indirect immunofluorescence was conducted as described in detail previously (Bojang *et al*., 2013).

**Table 2:** L1‐ORF1/2 TaqMan primers

Sequence name	Sequence
L1‐ORF1 Taqm ‐1F	AAT GTT AAG GGC AGC CAG AG
L1‐ORF1 Taqm ‐1R	CTC TTC TGG CTT GTA GGG TTT C
L1‐ORF1 Taqm PRB‐1	/56‐FAM/AC CCT CAA A/ZEN/G GAA AGC CCA TCA GA/3IABkFQ/
L1‐ORF1 Taqm ‐2F	CCT CAC CAG CAA CAG AAC AA
L1‐ORF1 Taqm ‐2R	CCT CCC GTA GCT CAG AGT AAT
L1‐ORF1 Taqm PRB‐2	/56‐FAM/TT GAC GAG C/ZEN/T GAG AGA AGA AGG CT/3IABkFQ/
L1‐ORF2 Taqm ‐1F	CTC AGA CCA CAG TGC AAT CA
L1‐ORF2 Taqm ‐1R	GTC ATT CAG GAG CAG GTT GT
L1‐ORF2 Taqm PRB‐1	/56‐FAM/AG CCG CTC A/ZEN/A CTA CAT GGA AAC TGA/3IABkFQ/

**Table 3:** L1‐ORF1/2 SYBR Green Primers

Sequence name	Sequence
L1‐ORF1‐1F	CCA AGTTGGAAAACACTCTGC
L1‐ORF1‐1R	TGTGGCGTTCTCTGTATTTCC
L1‐ORF2‐1F	TCGACACATACACTCTCCCAAG
L1‐ORF2‐1R	TGGTCCTGGACTCTTTTTGG

### Chromatin immunoprecipitation assay followed by RT‐PCR

Approximately 1 × 10^6^ HBECs were grown to 70% confluence and treated with 1% formaldehyde for 10 min at 37 °C and washed 2x with ice‐cold PBS supplemented with protease and phosphatase inhibitor cocktails (Cat#: 539‐134‐1SET). Chromatin immunoprecipitation assay (ChIP) was conducted as described before (Montoya‐Durango *et al*., 2009; Teneng *et al*., 2011). After incubation with respective antibodies for NuRD components, complexes were pelleted, the supernatant removed, and samples kept on ice. Immune complexes were eluted by addition of 250 μL elution buffer (1% SDS 0.1 M NaHCO_3_), vortexed briefly, and incubated at room temperature for 15 min with gentle rotation. The eluate was combined with 20 μL of 5M NaCl and incubated at 65 °C for 4 h to reverse formaldehyde cross‐linking of DNA/protein complexes. DNA was recovered by phenol/chloroform extraction and ethanol precipitation. DNA was resuspended in nuclease‐free H_2_O and quantified by Cytation 3 machine. Serial dilutions were completed to confirm a linear relationship while accounting for proper amplification of template to ensure equal amounts of starting template for all samples. RT‐PCR was used to determine enrichment for *L1*‐5′UTR sequences using tiling primers (P1‐P4) that span the 5′UTR promoter region of *L1*. The specificity of NuRD target recognition was examined in previous studies (Montoya‐Durango *et al*., 2009) and validated by DNA sequencing (Table 4 and Fig. S3). ChIP measurements in BaP‐ and DMSO‐treated samples used the P3 primer to focus on the RB/E2F recognition site within the promoter.

**Table 4:** PRIMERS FOR NuRD component used in RT‐PCR

Sequence name	Sequence
HCHD4‐1F	CTC GGG CCA GTG TAG AGG TC
HCHD4‐1R	TCT TCA TTT TCT GGG TGG GGT
HMTA2‐1F	GGC ATT ATG GTG AAA CGG GC
HMTA2‐1R	GGG CAT CAG CTG GGT TTA GT
HMTA3‐1F	AGT TGA GGC TGA CTT GAC CG
HMTA3‐1R	TCC CCT GAT ATG TGT TGC GG
HMBD2‐1F	CAA CCG GTA ACC AAA GTC ACA
HMBD2‐1R	GCT GAC GTG GCT GTT CAT TC
HMBD3‐1F	AAG ATG AAC AAG AGC CGC CA
HMBD3‐1R	TGG TAA TCT TGG TCA CCG GC
ACTIN‐1F	CCGGACGCTGAGTATGTTCA
ACTIN‐1R	TCTCCTGTCCTTCAGCCACTCT
18S‐1F	AGTCCCTGCCCTTTGTACACA
18S‐1R	GATCCGAGGGCCTCACTAAAC

### Formaldehyde‐assisted isolation of regulatory elements

Formaldehyde‐assisted isolation of regulatory elements (FAIRE) was used to study *L1* sequences associated with regulatory activity based on preferential formaldehyde cross‐linking of nucleosome‐bound DNA relative to nucleosome‐depleted regions of the genome (Giresi *et al*., 2007). HBECs were grown to 70% confluence and treated with 0.1 μm BaP or equivalent volume of DMSO for 12 h. The medium was removed and the cells cross‐linked with 1% formaldehyde for 10 min at 37 °C. Cells were washed 2X with ice‐cold DPBS (Dulbecco's phosphate‐buffered saline pH 7.4) supplemented with protease and phosphatase inhibitor cocktails (Cat#: 539‐134‐1SET) before being scraped and pelleted for 5 min at 800 ***g*** at 4 °C and resuspended in DPBS. Two 500‐μL aliquots of lysate were set aside for RNA analysis and input control. A total of 500 μL of lysate was resuspended in FAIRE lysis buffer (1% SDS, 100 mm NaCL, 50 mm Tris pH 8, 2 mm EDTA, and 2 mm EGTA supplemented with protease and phosphatase inhibitor cocktails) and sonicated as described for 30 sec and cooled in ice for ~5 min. Unbound DNA and DNA cross‐linked to proteins were separated by phenol/chloroform extraction. Unbound DNA in the aqueous phase was ethanol precipitated with 1/10 of 3M sodium acetate (NaAc) and 2.5X of 95% ethanol (ETOH) overnight followed by centrifugation at 14000 g for 20 min. DNA was washed with 70% ETOH once, air‐dried, and resuspended in 50 μL of sterile nuclease‐free H_2_O. Total DNA was quantified and serial dilutions of 2.5, 5, and 10 ng used for RT‐PCR analysis using a primer set (P4) that spans the CpG shore region of the *L1* promoter (Table 5). For *L1* transcript analysis, RNA was isolated as previously described using RNeasy Plus Mini Kit (Cat#: 74134). Total gDNA for use as input control was isolated using Purelink genomic DNA isolation kit (Cat#: K1820‐01).

**Table 5:** Mi2β ChIP RT‐PCR primers

Sequence name	Sequence
HL1UTR‐1f	GGAACAGCTCCGGTCTACAG
HL1UTR‐1R	TCACCCCTTTCTTTGACTCG
HL1UTR‐2f	GAGTTCCCTTTCCGAGTCAA
HL1UTR‐2R	TGTGCTAGCAATCAGCGAGA
HL1UTR‐3f	CACGGAATCTCGCTGATTG
HL1UTR‐3R	CCAGAGGTGGAGCCTACAGA
HL1UTR‐4f	CAGGGAAGCTCGAACTGG
HL1UTR‐4R	AGCTGCAGGTCTGTTGGAAT

### Viral transfection and luciferase analysis

HEK293T‐17 cells were used to generate virus titers for subsequent transduction of the virus containing Mi2β and mutant plasmids into BEAS‐2B cells. HEK293T‐17 cells were grown to 90% confluence and transfected with Mi2βWT/mutants retroviral vectors (6 μg), combined with pSPAX‐2 packaging vector (3 μg) and pVSV‐G envelope vector (1.5 μg) diluted in 500 μL of dilution media (10 mm HEPES/150 mm NaCl, pH 7.05). Retroviral packaging vectors (pSPAX‐2 and pVSV‐G) were a kind gift from Schenten at the University of Arizona (Addgene plasmid # 12260 and Addgene plasmid # 36399, respectively). An aliquot of 42 μL of PEI (1.0 μg/μL) was added to each transfection and incubated at room temperature for 15 min. DNA/PEI complexes were added to the cells and incubated for 18 h. Transfection media were removed and new media containing 10% FBS added. The viral supernatant was collected the next day and filtered through 0.45‐μm PES filter tubes. Viral titer was incubated with one volume of X‐concentrator (Clontech) overnight and centrifuged at 1500 g for 45 min. The supernatant was removed and virus resuspended in 100 μL PBS to make 10X viral concentrate. A total of 10 μL of viral titer was used to transiently transduce transfected cells. HBECs were grown to 70% confluence and transiently transfected with 0.75 μg of luciferase and 0.25 μg of Renilla vectors. Transfection media were removed 18 h later. Cells were allowed to recover for 24 h and then transduced with Mi2βWT or mutant retroviral vectors or cotreated with BaP and retroviral vectors for 24 h. Cells were lysed passively for 15 min at room temperature and luciferase and Renilla luminescence measured using a Cytation 3 machine. Relative luminescence units (RLU) were calculated as a ratio of firefly luciferase to Renilla.

### Mouse xenografts and immunohistochemistry

HBECs (6 × 10^6^) cells stably expressing control (pB001^ctr^) or wild‐type (WT) *L1* plasmids (pB015^wt^) were mixed with Matrigel and injected subcutaneously into anesthetized SCID mice at the University of Arizona Cancer Center Core Facility. Mouse weights and tumor burden were monitored twice weekly for up to 90 days, with tumor volumes estimated using the following formula: [(width)^2 ^× length]/2. A 1 : 50 dilution of ORF1p antibody was used to monitor L1‐ORF1 protein in tumors derived from grafted cells or human tissues by immunohistochemistry (IHC). Images were taken using an Axiovision inverted microscope at 40 ×  magnification.

### Statistics

The differences between groups were evaluated using the Mann–Whitney U‐test to compare samples drawn from the same distribution. Mann–Whitney U‐test is a nonparametric alternative test often used to evaluate normalized data because it does not require the assumption of normal distribution when trying to determine differences between two independent samples. The number of replicates and independent measures for each of experiment are included in the figure legends.

## Results

### *L1* reactivation by BaP disrupts NuRD promoter occupancy and partially shifts constituent proteins from the nuclear to cytoplasmic compartment

We have previously shown that the silenced *L1‐Ta* promoter is enriched in repressive heterochromatin marks, namely histone‐3 lysine‐9 trimethylation (H3K9Me3) and histone‐4 lysine‐20 trimethylation (H4K20Me3) (Teneng *et al*., 2011). We also showed that RB proteins stabilize the NuRD multiprotein complex to effect *L1* silencing and that BaP interferes with RB functions via AHR‐dependent mechanisms (Montoya‐Durango *et al*., 2009, 2016; Teneng *et al*., 2007). AHR is present in the repressor complex assembled by RB on the *L1* promoter in human and murine cells (Montoya‐Durango *et al*., 2009). However, the structural and functional determinants of NuRD corepression during *L1* reactivation by tobacco carcinogens in HBECs have not been elucidated. To fill this gap, experiments were initiated to study *L1* reactivation in HBECs challenged with BaP, a carcinogenic aromatic hydrocarbon present in tobacco smoke.

**Figure 1:**
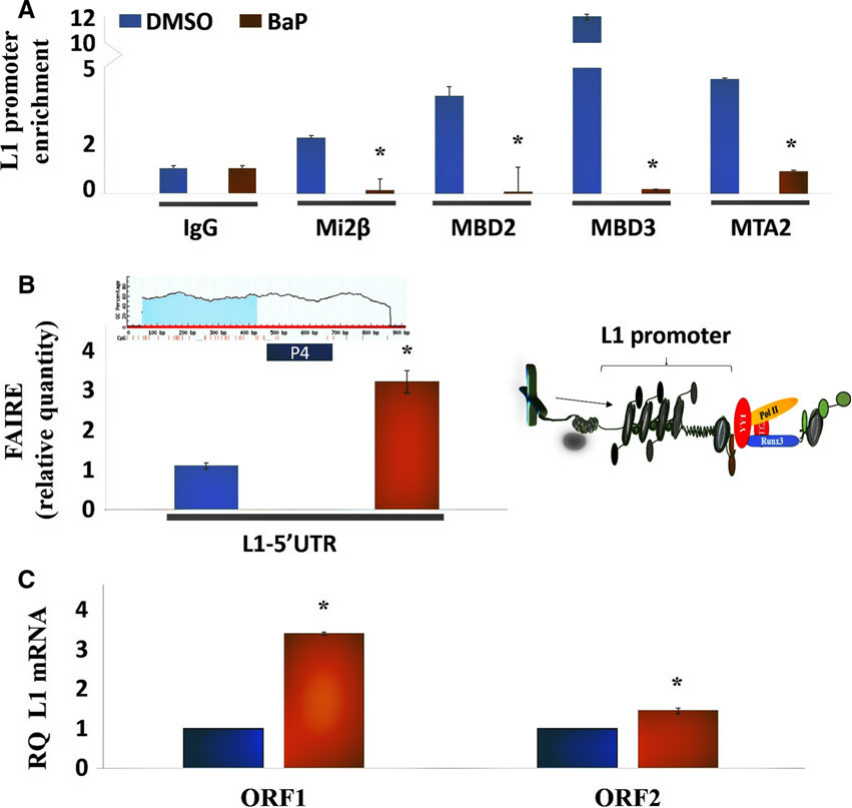
Displacement of NuRD macromolecular complex constituents from the *L1* promoter by BaP shifts *L1* sequences from heterochromatin to euchromatin and leads to transcriptional activation. (A) ChIP experiments of L1 promoter using primer 3 in HBECs treated with DMSO control or 0.1 μm BaP for 12 h. Fold enrichment was calculated relative to IgG control. Each assay was performed at least three times in triplicate, and the values shown are representative of combined experiments. Primer sequences are provided in Table**** 4. (B) FAIRE analysis of HBECs under DMSO control or BaP‐treated conditions using primer 4 (P4) to examine protein‐bound versus unbound *L1* DNA sequences. Similar results were seen in three independent experiments using triplicates in each of these experiments. (C) RT‐PCR analysis of ORF1 and ORF2 in DMSO control or BaP‐treated cells. Similar results were seen in three independent experiments using triplicates in each experiment. Primer sequences are provided in Tables 2 and 3. RQ, relative quantity. Differences between groups were evaluated by the Mann–Whitney *U*‐test to compare samples from the same distribution. Statistical significance is denoted by (*).

The retention of NuRD constituent proteins on the *L1*‐5′UTR promoter in HBECs challenged with 0.1 μm BaP is presented in Fig. 1A. This concentration was chosen based on dose–response and time–response experiments in BEAS 2B cells showing significant induction of *L1* in the range of 0.1 to 3 μm (data not shown). Marked reductions in enrichment for Mi2β, MBD2/3, and MTA2 were seen 12 h after carcinogen challenge, indicating that BaP displaces NuRD macromolecular complex constituents from the *L1* promoter. Next, FAIRE was employed to separate unbound and bound DNA sequences based on their relative affinities for the aqueous and organic phases, respectively (Giresi *et al*., 2007). BaP treatment reduced protein trapping at genomic regions containing *L1*‐5′UTR DNA sequence (Fig. 1B), indicating a shift of *L1* chromatin from a heterochromatic to euchromatic state. These changes were associated with transcriptional activation and accumulation of *L1* ORF1 and ORF2 mRNA (Fig. 1C). The lower levels of ORF2 mRNA compared to ORF1 mRNA are consistent with the presence of cryptic polyadenylation sites in ORF2 that cause early termination of transcription (Kazazian *et al*., 1988).

**Figure 2:**
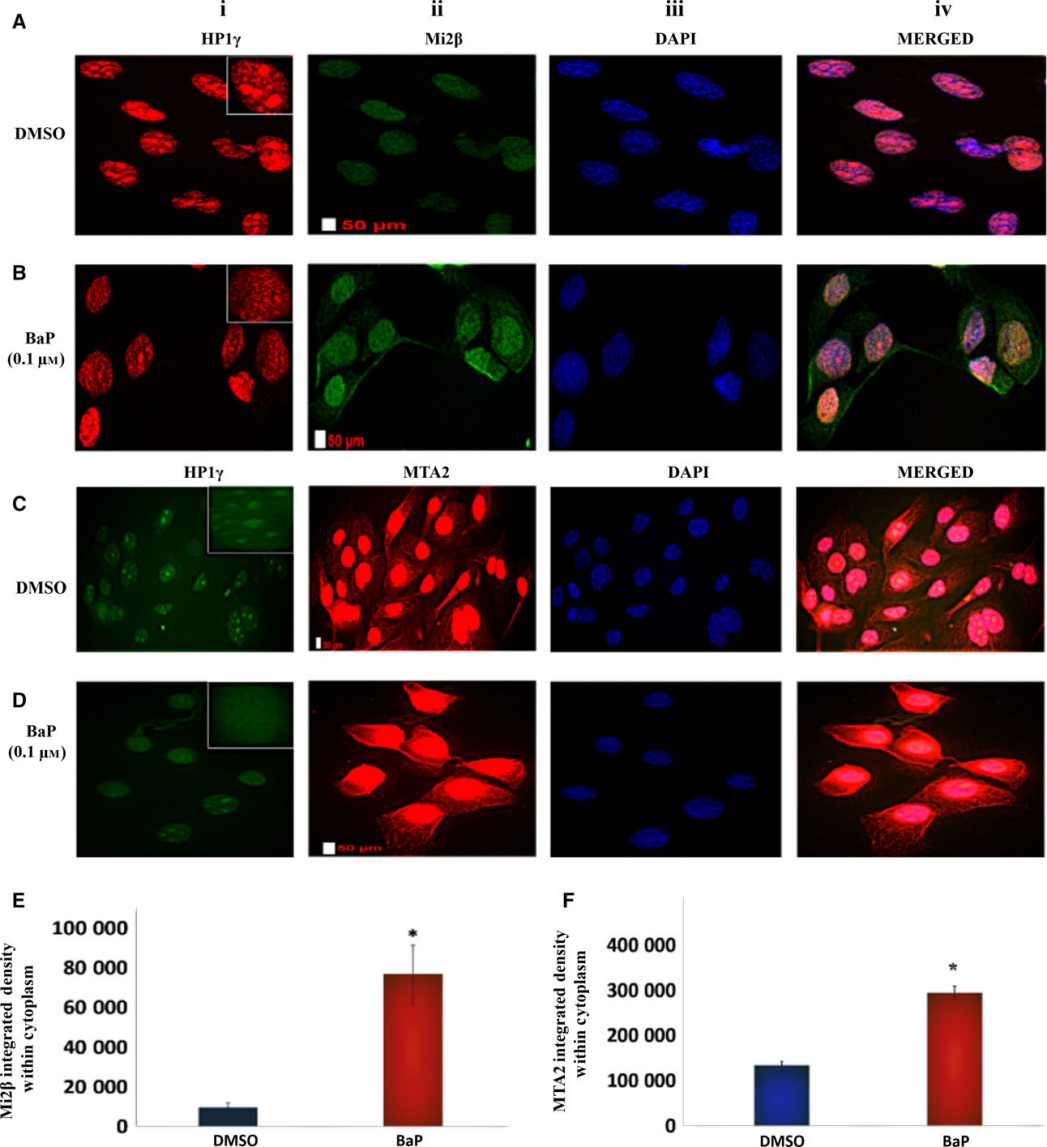
Benzo(a)pyrene disassembles the NuRD complex and shifts constituent proteins from the nuclear to cytoplasmic compartment. (A, B) Mi2β and (C, D) MTA2 immunofluorescence staining in HBECs treated with DMSO or 0.1 μm BaP for 12 h. (E, F) Densitometric measurement of fluorescence intensity for Mi2β and MTA2 in the cytoplasmic compartment of control and BaP‐treated HBECs, respectively. HP1γ was used as an index of structural integrity of heterochromatin domains. DAPI was used to stain the nuclei, with merged signals denoting colocalization of proteins. The staining for HP1γ was switched from red to green and vice versa to control for nonspecific staining by the secondary antibody. Similar results were seen in three independent experiments. The differences between groups were evaluated by the Mann–Whitney *U*‐test to compare samples drawn from the same distribution (*n* = 32 cells/experiment). Scale bars are 50 μm. Statistical significance is denoted by (*).

*L1* reactivation in HBECs following carcinogen challenge showed time‐dependent profiles (Fig. S2), with induction of *L1* mRNA peaking at 12 h and returning to baseline by 48 h. Western analysis at 12 h showed accumulation of *L1* ORF1 and ORF2 proteins in cells challenged with BaP (Fig. S2), confirming effective translation of mRNA into proteins. CYP1A1 protein was detected by immunofluorescence in BEAS 2B cells (Fig. S4), suggesting that BaP metabolites may contribute to the *L1* induction response. We have previously shown that CYP‐derived metabolites of BaP readily activate *L1* and are significantly more potent than the parent compound in vascular cells (Lu and Ramos, 2003). To further evaluate the disruption of NuRD corepressor complex interactions by BaP, the localization of NuRD constituent proteins was examined by indirect immunofluorescence. In these studies, HP1γ staining was measured as an index of the structural integrity of heterochromatin domains (Lechner *et al*., 2000; Mansfield *et al*., 2011). HP1γ staining in vehicle‐treated HBECs showed distinct focal accumulations, while staining in BaP‐treated cells was diffuse and considerably less pronounced (Fig. 2A,D, column 1). Mi2β and MTA2 staining strongly colocalized with DAPI staining, and was visibly nuclear in DMSO‐treated cells, indicating that under basal conditions these proteins preferentially reside within the nuclear compartment (Fig. 2A,C, columns ii and iv). In keeping with previous reports (Takeshita *et al*., 2016), the nuclei of BaP‐treated cells appeared swollen at times. Staining for Mi2β and MTA2 in HBECs treated with BaP was distributed between the nuclear and cytoplasmic compartments (Fig. 2B,D, columns ii and iv), suggesting a partial shift to the cytoplasmic compartment. To confirm this interpretation, nuclear fluorescence intensity was quantified and subtracted from total fluorescence intensity using imagej software (imagej.nih.gov). Increased fluorescence intensity for cytoplasmic Mi2β and MTA2 was observed in cells treated with BaP compared to DMSO controls (Fig. 2E,F, respectively). Thus, challenge of HBECs with BaP is associated with disintegration of heterochromatin leading to disassembly and detachment of NuRD components from the *L1* promoter. These changes in turn were associated with a partial shift of constituent proteins from the nuclear to cytoplasmic compartment. The exact mechanism responsible for translocation of NuRD constituent proteins is not known, but may involve nuclear export signals and exportins, as shown previously for MBD2‐interacting proteins (Lechner *et al*., 2000).

### Mi2β binding to the *L1* promoter in HBECs effects *L1* retroelement silencing

**Figure 3:**
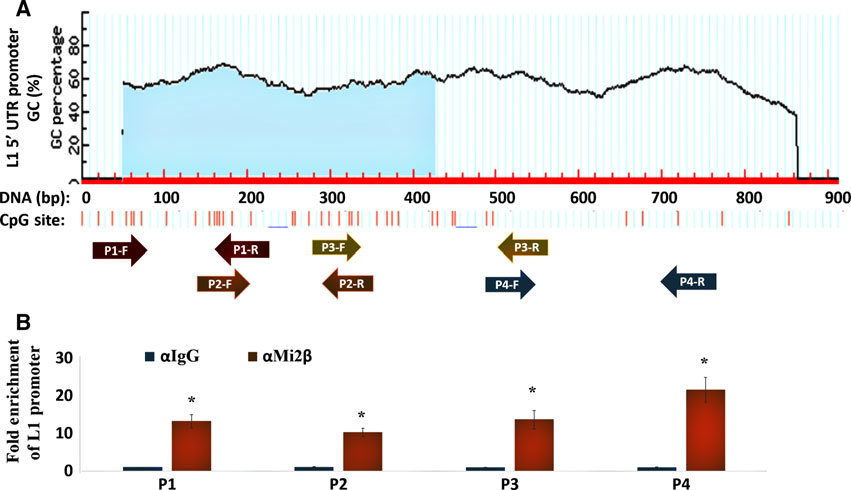
Mi2β Interacts with *L1* Promoter Sequences. (A) Schematic representation of the location of tiling primers used to characterize Mi2β binding along the full‐length *L1*‐5′UTR (Table 4). CpG islands and shores within the promoter are also shown. (B) Solubilized chromatin was immunoprecipitated with antibodies against Mi2β or mouse IgG. After isolation and normalization of genomic DNA, RT‐PCR was performed using tiling primers specific to *L1*‐5′UTR promoter. Similar results were seen in three independent experiments. Statistical significance is denoted by (*).

Mi2β is an ATP‐dependent helicase subunit with nucleosome remodeling activity that is specific to the NuRD complex. Therefore, the next set of experiments was completed to characterize the influence of Mi2β binding to the *L1* promoter in HBECs by ChIP using tiling primers (P1–P4) that spanned the complete *L1‐Ta* 5′UTR promoter sequence coupled with antibodies specific to Mi2β (Fig. 3A). *L1‐Ta* 5′UTR sequences were highly enriched in samples treated with Mi2β antibody compared to IgG control for all primer sets used to amplify promoter sequences (Fig. 3B). DNA electrophoresis and sequencing were completed to confirm the identity and size (P1: 224 bp, P2: 175 bp, P3: 188 bp, and P4: 315 bp) of amplified sequences (Fig. S3). Thus, chromatin‐bound Mi2β is present across the entire length of the *L1* promoter.

**Figure 4:**
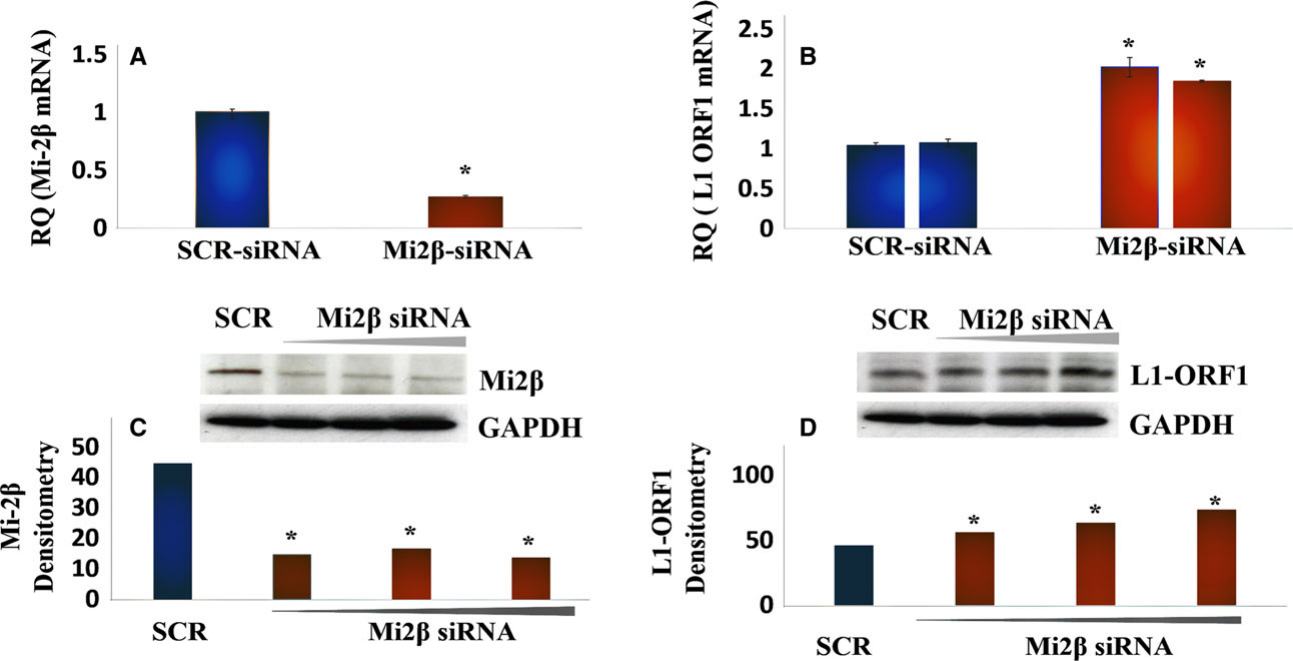
Mi2β Binding to *L1* Promoter Sequences Effects L1 Retroelement Silencing. (A) RT‐PCR analysis of cells treated with scrambled (SCR) siRNA or siRNA against M12β. Similar results were seen in three independent experiments using triplicates in each of these experiments. (B) RT‐PCR analysis of *L1* mRNA levels in cells treated with scrambled siRNA or siRNA against M12β. Similar results were seen in three independent experiments using triplicates in each of these experiments. (C) Western blot analysis of Mi2β levels following siRNA silencing. Similar results were seen in three independent experiments. (D) Western blot analysis of *L1* ORF1p following Mi2β silencing. The image showed the same membrane probed for Mi2β, *L1* ORF1p, and GAPDH. Similar results were seen in three independent experiments. Differences between groups were evaluated by the Mann–Whitney *U*‐test to compare samples from the same distribution. Statistical significance is denoted by (*).

**Figure 5:**
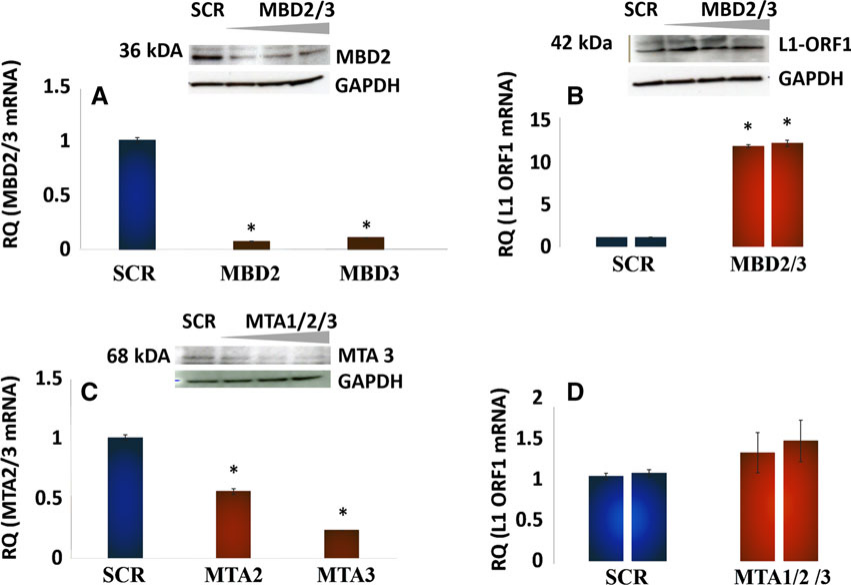
MBD2/3, but not MTA1/2/3, also participates in *L1* Retroelement Silencing. (A) Western blot for MBD2 and RT‐PCR analysis of MBD2/3 in HBECs treated with scrambled siRNA or siRNA against MBD2/3. Similar results were seen in three independent experiments using triplicates in each of these experiments. (B) *L1* ORF1p mRNA and protein in HBECs treated with scrambled siRNA or siRNA against MBD2/3. Similar results were seen in three independent experiments. (C) Western blot for MTA3 and RT‐PCR analysis of MTA2/3 in HBECs treated with scrambled siRNA or siRNA against MTA1/2/3. Similar results were seen in three independent experiments using triplicates in each of these experiments. (D) *L1* ORF1p mRNA in HBECs treated with scrambled siRNA or siRNA against MTA1/2/3. Similar results were seen in three independent experiments. The primer sequences used are presented as supplementary data in tabular form. Differences between groups were evaluated by the Mann–Whitney *U*‐test to compare samples from the same distribution. Statistical significance is denoted by (*).

Genetic ablation of Mi2α/β mRNA and protein using target‐specific siRNA (Fig. 4A, panels i and iii, respectively) increased the levels of *L1* ORF1 mRNA and protein (Fig. 4A, panels ii and iv, respectively). *L1* ORF1 mRNA and protein were detectable under unstimulated conditions (See Fig. S1) and significantly upregulated by siRNA treatment. The functional role of MBD2/3 and MTA2/3 was examined next using gene‐specific siRNA directed against these proteins (Fig. 5A,B). *L1*‐ORF1 mRNA and protein were markedly upregulated by MBD2/3 knockdown (Fig. 5A, panel ii), while ablation of MTAs was without effect (Fig. 5B, panel ii). These findings identify Mi2β and MBDs as the key architectural elements that effect *L1* silencing. The negative regulatory function of Mi2β likely reflects its activity within the NuRD macromolecular complex, while MBDs may exert regulatory control through binding to methyl groups in DNA. Collectively, these findings show that functional modulation of Mi2β and MBDs is a fundamental molecular event in the activation of *L1* in HBECs.

### Mutations in the DNA‐ and ATPase‐binding domains, but not the chromodomain of Mi2β negatively regulate NuRD corepressor activity

**Figure 6:**
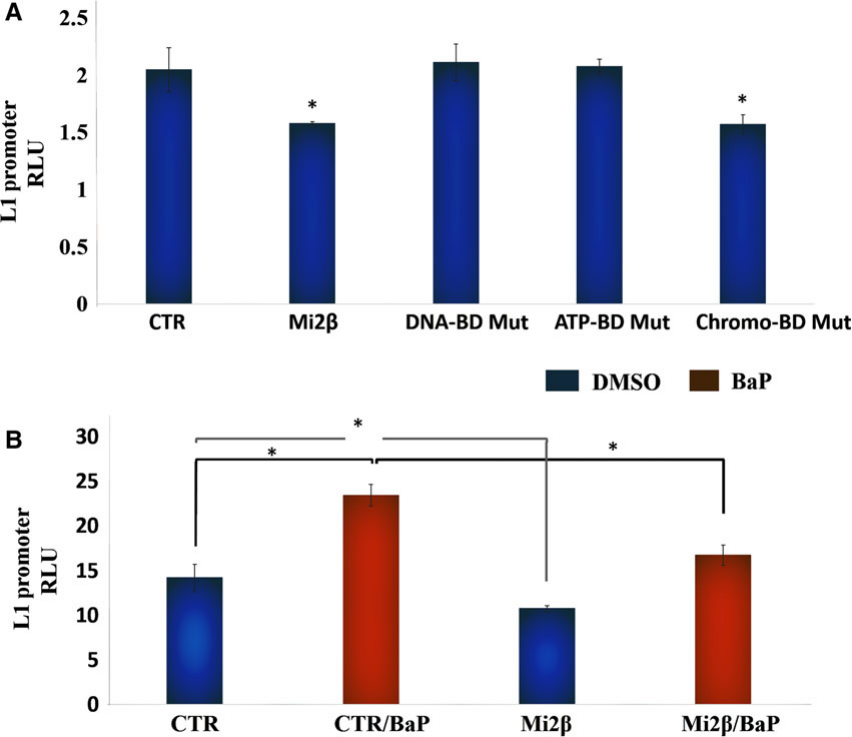
The DNA and ATPase domains, but not the chromodomain, are necessary for *L1* promoter silencing. (A) Mi2β and mutant retroviral vectors (i.e., W508A chromodomain mutant, E874A DNA‐binding mutant and W879A ATPase domain mutant) were transduced into HBECs transiently transfected with *L1*‐5′UTR promoter‐driven luciferase and Renilla vector as internal control. RLU values were calculated as a ratio of luciferase luminescence over Renilla luminescence. Similar results were seen in three independent experiments using triplicates in each of these experiments. (B) Influence of Mi2β on L1 Reactivation by BaP. Cotreatment of HBECs with BaP or DMSO control with Mi2βWT retroviral vectors in cells transiently transfected with *L1‐*5′UTR promoter‐driven luciferase gene. Control (CTR) consisted of pSPAX‐2 packaging vector and pVSV‐G envelope vector. Transfection efficiency was controlled by Renilla vector. Similar results were seen in three independent experiments using triplicates for each of these experiments. Differences between groups were evaluated by the Mann–Whitney U‐test to compare samples from the same distribution. Statistical significance is denoted by (*).

To further confirm the role Mi2β in *L1* suppression, the suppressor activity of Mi2βWT was compared to that of mutants within the DNA‐binding domain, ATPase‐binding domain, and chromodomain (Ramírez *et al*., 2012). *L1‐Ta* 5′UTR promoter‐driven luciferase and Renilla vector (internal control) were transiently transfected into HBECs. Cells recovered from transfection for 24 h before treatment with retroviral vectors encoding Mi2βWT or mutants for an additional 24 h. Transduction efficiency was optimal 24 h after exposure (data not shown). A significant, but modest, reduction in *L1* reporter activity was seen in Mi2β WT‐transfected cells (Fig. 6A). The modest response likely implicates significant contributions by other accessory proteins to basal activation of the *L1* promoter. Importantly, mutants in the DNA‐ and ATPase‐binding domains of Mi2β lost their ability to suppress luciferase reporter gene expression, while the chromodomain mutant was without effect (Fig. 6A). Cotreatment of cells transfected with Mi2βWT retroviral vectors significantly reduced both basal‐ and BaP‐driven luciferase expression (Fig. 6B), confirming that disruption of Mi2β activity by BaP plays an important role in regulating the repressive functions of the NuRD complex on *L1*. The partial reversal seen upon reconstitution with Mi2β confirms that combinatorial functions with other members of the repressor complex are essential for *L1* repression.

### Expression of a constitutively active synthetic *L1* is oncogenic in a SCID xenograft mouse model

**Figure 7:**
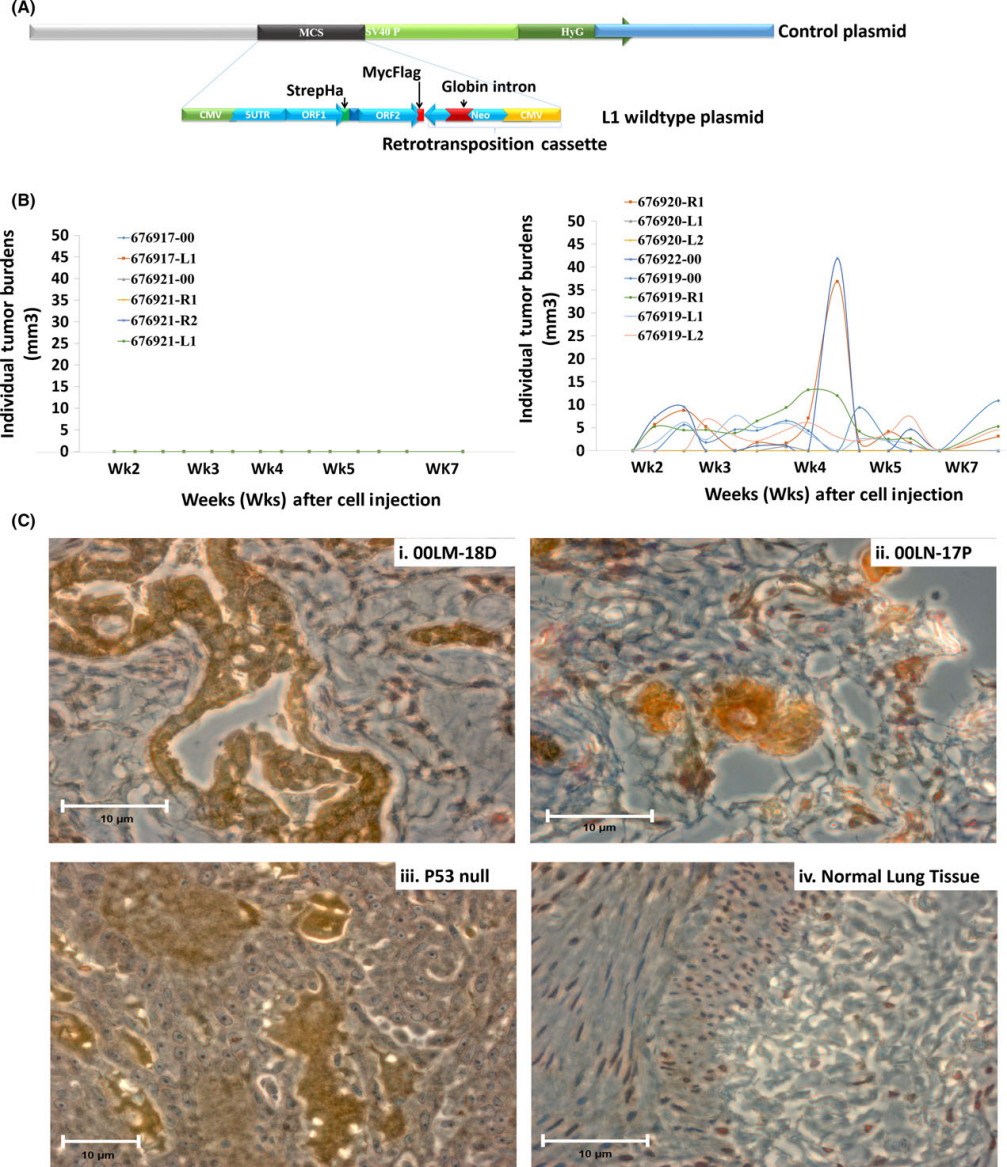
Oncogenicity of Constitutively Active *L1* in Human Bronchial Epithelial Cells. (A) Schematic representation of the construct utilized for stable expression of wild‐type L1 or empty vector. (B) Tumor burden in SCID mice injected once subcutaneously with control (*n* = 6) or wild‐type L1 (*n* = 15) vectors. (C) Immunohistochemistry of L1‐ORF1p staining in (i) tumors isolated from 00LM‐18 and (ii) 00LN‐17P, SCID mice (iii) p53‐null lung tissue, (iv) normal lung tissue. Scaled bars represent 10 μm.

The final set of experiments examined the oncogenic potential of *L1* in a SCID mouse xenograft model. Given that *L1* activation in HBECs by BaP peaks at 12 h and returns to baseline by 48 h (Fig. S2), an *in vitro* model of constitutive *L1* activation that replicates the smoking behavior of habitual users who smoke ≥12 cigarettes per day was examined. Our estimation is consistent with previous reports showing regular patterns of smoking frequency of smokers (Chandra *et al*., 2007). SCID mice were injected subcutaneously with 6 × 10^6^ HBECs stably expressing empty pB001^ctr^ vector (*n* = 6) or a constitutively active wild‐type *L1* construct (pB015^WT^) (*n* = 15) (Bojang *et al*., 2013). pB015^WT^ consists of *L1* ^RP^ sequence cloned into a pGL4.15 vector backbone with *L1* ORF1 and ORF2 proteins tagged with streptavidin–hemagglutinin (Strep–HA) and flag‐myc double tags, respectively (Fig. 7A). A cytomegalovirus (CMV) promoter was placed upstream of the *L1*‐5′UTR for constitutive expression. No differences in animal weights were seen between the two groups (data not shown). *L1* was tumorigenic in SCID mice, with tumor burden first detected one week after inoculation and sustained for up to eight weeks (Fig. 7B). No tumors were seen in control mice at any time during the experiment, confirming the nontumorigenic phenotype of HBECs (i.e., BEAS‐2B cells), and the innocuous nature of the control vector. Peak tumor incidence occurred four weeks after inoculation (12/15 mice), with significant tumor growths ranging in size from 1.47 to 42.0 mm^2^ in 6 of 15 mice during week five. Variable degrees of tumor regression and recurrence were seen over the course of the eight‐week experiment (Fig. 7B). The expression of *L1*‐ORF1 protein was examined in the grafted tumors by IHC to confirm molecular phenotype (Fig. 7C). *L1*‐ORF1 protein expression was positive in all mouse tumors examined (*n* = 6). Representative samples are shown in Fig. 7C, panels i and ii. Normal human lung tissue and p53 null lung tissue were used as negative and positive controls, respectively (Fig. 7C, panels iii and iv). High levels of *L1*‐ORF1 protein were detected in p53 null tissue, but not in normal lung. These findings demonstrate that activation of *L1* in HBECs creates a microenvironment that supports oncogenic transformation and implicate molecular interactions between *L1* and p53 as critical players in lung cancer.

## Discussion

Evidence is presented here establishing a functional linkage between NuRD macromolecular complex disassembly, *L1*‐*Ta* reactivation, and malignant transformation in HBECs challenged with the lung carcinogen BaP. These findings build upon earlier reports by our group showing that *L1* reactivation involves the following: 1) enrichment of activating H3K4Me3 and H3K9Ac marks; 2) increased histone H3 acetylation of the 5′UTR; 3) reduced DNMT1 recruitment; and 4) CpG hypomethylation in both transformed and nontransformed epithelial cells (Montoya‐Durango *et al*., 2009; Teneng *et al*., 2011). Thus, the association of NuRD complexes with the *L1* promoter may serve as a key regulatory signal for the formation of regional domains of heterochromatin that can be disrupted by the lung carcinogen BaP during the course of malignant progression. NuRD subunits are known to associate with oncogenic transcription factors, and the loss of NuRD function has been linked to various types of malignancies (Lai and Wade, 2011). Furthermore, Mi2β binds to H3K9Me3, a modification characteristic of the silenced *L1* promoter in transformed cells (Mansfield *et al*., 2011; Teneng *et al*., 2011), and activating histone marks delocalize Mi2β and MDBs and disrupt architectural anchoring of the NuRD complex to DNA (Allen *et al*., 2013; Baubec *et al*., 2013).

The ability of BaP to interfere with the assembly of NuRD subunits and their function likely involves AHR, a ligand‐activated transcription factor regulated by BaP during the course of *L1* reactivation (Teneng *et al*., 2007). AHR was first identified as the nuclear receptor responsible for transcriptional regulation of cytochromes P450 by dioxin and related aromatic hydrocarbons, and later shown to bind a variety of endogenous and exogenous ligands to effect molecular control of proliferation, apoptosis, differentiation, adhesion, and pluripotency. We have previously shown that AHR is present in the repressor complex assembled by RB on the *L1* promoter in human and murine cells (Montoya‐Durango *et al*., 2009). Others have shown that AHR interacts with NuRD complex subunits to regulate differentiation‐specific genes that interfere with early embryonic programming (Gialitakis *et al*., 2017). Thus, AHR may be the critical effector used by BaP to interfere with NuRD functions in HBECs. This suggestion is supported by our own studies showing that genetic ablation of AHR under basal conditions in BEAS‐2B cells dramatically increases *L1* mRNA levels (Bojang and Ramos, unpublished data). As such, constitutive AHR may support critical protein–protein interactions that are disrupted in the presence of BaP to facilitate disassembly of the NuRD repressor complex. Furthermore, AHR interacts with TGF‐β1, an upstream regulator of *L1* and inducer of EMT in HBECs (Chang *et al*., 2007; Ramos *et al*., 2011; Reyes‐Reyes *et al*., 2017). Thus, structural and functional interactions between AHR and NuRD complex subunits may cooperate with TGF‐β1 in a highly contextual manner to regulate *L1*. Wang *et al*. (2009) reported that NuRD complexes regulate TGF‐β1 signaling by enlisting LSD1, a lysine‐specific demethylase that affords histone demethylase activity to the chromatin remodeling capacity of the NuRD complex (Wang *et al*., 2009). AHR may also participate in the regulation of CYP genes involved in BaP metabolism, a suggestion consistent with the expression of CYP1A1 protein in BEAS‐2B cells and our previous report that oxidative BaP metabolites activate *L1* expression. Although the relative contributions of unmetabolized versus metabolized BaP to the tumorigenic response in BEAS‐2B cells were not examined, the 12‐h lag time for induction of *L1* in BEAS‐2B cells suggests that metabolism of the parent hydrocarbon may contribute to the *L1* reactivation response.

Based on our collective findings, we conclude that combinatorial interactions between AHR, RB, p53, and NuRD subunits effect epigenetic silencing of *L1* and are direct targets of the lung carcinogen BaP during the course of *L1* reactivation and malignant transformation (Lai and Wade, 2011; Montoya‐Durango *et al*., 2009; Teneng *et al*., 2007). The immortalization of BEAS‐2B cells with SV40 large T‐antigen likely sensitizes RB and p53 to create a permissive environment for *L1* reactivation by the carcinogen. This is consistent with the appearance of *L1* protein in p53‐null lung cancer, but not normal tissue, and the exquisite sensitivity of BEAS‐2B cells to BaP. However, despite SV40 immortalization, the BEAS‐2B cell line is not tumorigenic in the absence of *L1* or carcinogen, remains RB and p53 competent during serial culture, and retains intact Akt signaling (Pan *et al*., 2011). We previously showed that forced expression of *L1* in embryonic kidney cells disrupts differentiation programming and alters growth factor responsiveness (Ramos *et al*., 2011), suggesting that *L1* alone is sufficient to drive the appearance of oncogenic phenotypes. NuRD complex disassembly has been linked to malignant progression through disruption of cellular differentiation and transformation (Kaur *et al*., 2007), while *L1* reactivation has been linked with genomewide reprogramming, EMT, malignant transformation, and poor prognosis in NSCLC patients (Bojang *et al*., 2013; Ikeda *et al*., 2013; Saito *et al*., 2010). Further, disassembly of the NuRD complex from promoters is known to recruit SWI/SNF complexes that regulate EMT (Mohd‐Sarip *et al*., 2017). The reactivation and mobilization of *L1* can predispose lung cancer genomes to aberrant epigenetic control by creating sloppy DNA methylation shores that influence the methylation status and expression of surrounding loci (Grandi *et al*., 2015). In accord with this view, *de novo* somatic *L1* retrotransposition occurs at higher frequencies in NSCLCs than in other tumors, and site‐specific hypomethylation of *L1*s is associated with pathological features of NSCLC patients (Ikeda *et al*., 2013; Saito *et al*., 2010). Thus, the carcinogenicity of BaP and its impact on NSCLC evolution are likely tied to the disruption of NuRD complex functions and *L1* reactivation.

While the repressive role of the NuRD complex has been extensively studied, it is not yet known how the loss of one or more of its components affects overall function. Some of the components have been shown to be mutually exclusive, while others exert opposing functions (Denslow and Wade, 2007). The results of our siRNA analyses showed that loss of Mi2β and MBD2/3 increases *L1* mRNA, while loss of MTAs was without effect. These results are in line with the organization of the NuRD complex in a repressive state, where proteins are anchored to the DNA through Mi2β and MBD2/3, and MTAs do not directly contact DNA (Lai and Wade 2011). MTAs have been shown to interact with RBBP4/7 and GATAD2α/β, which in turn interact with MBD2/3 to anchor MTAs to the complex (Lai and Wade 2011; Allen *et al*., 2013). Therefore, promoter‐specific analysis would be required to determine the functional impact of NuRD‐related functions for other genetic targets, as well as its functionality in different cellular contexts.

Transcriptional regulation of *L1* is highly contextual, cell type specific, and dependent upon the activation stimulus (Teneng *et al*., 2007). Given the functional linkage between NuRD core proteins and *L1* retroelement reactivation, regulatory control of *L1* is likely coupled to the expression of accessory proteins involved in the regulation of NuRD core protein functions (Lai and Wade 2011; Allen *et al*., 2013). For instance, the interaction of HIF1α with NuRD activates transcription, while interactions between NuRD and p53 are repressive (Lai and Wade 2011; Allen *et al*., 2013). This is in keeping with our finding that functional interactions between RB and AHR proteins repress *L1* through interactions with the NuRD complex under basal conditions (Montoya‐Durango *et al*., 2009, 2016). Mi2β plays a prominent role in the regulation of *L1* expression upon carcinogen activation, with the DNA‐binding and ATPase enzymatic activities, but not the chromo protein–protein interaction domain, serving to interfere with ectopic *L1* expression. The minimal role of the chromodomain is consistent with the function and architecture of the NuRD complex, where NuRD core proteins mediate not only protein–protein interactions, but also remodeling of nucleosomes, histone deacetylation and demethylation, and interactions with methylated DNA (Baubec *et al*., 2013; Xue *et al*., 1998). Our data suggest that DNA methylation is a prerequisite for *L1* suppression, especially in the context of MBD functions (Jiang *et al*., 2004; Teneng *et al*., 2011). This assertion is consistent with the finding that cotreatment of HBECs with Mi2β and BaP did not completely abolish BaP‐driven luciferase activity.

Elevated expression of *L1* retrotransposon has been associated with oncogenic transformation and *L1* hypomethylation is known to induce stem cell‐like properties and EMT in NSCLCs (Ikeda *et al*., 2013; Saito *et al*., 2010). However, the contextual determinants that define *L1* functions as a driver or passenger event in tumorigenesis are not fully understood. Our *in vivo* studies examined these relationships using a constitutive model of *L1* activation in HBECs that mimics the repeated cycles of exposure seen in smokers who consume 12 or more cigarettes per day (Chandra *et al*., 2007; Nanez *et al*., 2011). Evidence was obtained that expression of constitutively active *L1* in HBECs is oncogenic in a SCID mouse xenograft model, confirming that *L1* can function as an initiating event during malignant transformation of lung epithelial cells. The oncogenic activity of *L1* may be exerted via retrotransposition‐dependent and retrotransposition‐independent mechanisms, with the former involving insertions, deletions, and structural damage, and the latter involving oncogenic signaling pathways that couple with EMT (Reyes‐Reyes *et al*., 2017).

Interestingly, tumor growth in mice grafted with cells expressing *L1* was restricted over time, suggesting that the oncogenic response initiated by *L1* cooperates with other pathways that sustain the malignant response. In this regard, the ability of BaP to damage DNA and to mutate both p53 and KRAS in HBECs may amplify the DNA damage response, propagate waves of genomic instability, and disrupt genetic regulatory networks of growth and differentiation. In fact, p53 is known to restrict retrotransposon activity through interaction with components of the piRNA (PIWI Interacting RNA) pathway (Wylie *et al*., 2016). Our own evidence supports this as *L1*‐encoded ORF‐1p was increased in human lung tumors null for p53, a finding consistent with previous reports (Wylie *et al*., 2016). We recently showed that a reverse transcriptase mutant of *L1* can induce tumors in athymic mice that outgrow those induced by wild‐type *L1* (Reyes‐Reyes *et al*., 2017), suggesting that retrotransposition and genotoxicity from the unregulated endonuclease activity of wild‐type *L1* pose significant restrictions on the tumorigenic process. This is an area worthy of exploration as novel chemotherapeutic agents can be designed to exploit this property in the clinical management of lung tumors.

In conclusion, evidence is presented here showing that challenge of HBECs with the lung carcinogen BaP shifts the *L1‐Ta* promoter from a heterochromatic to euchromatic state through disassembly of the NuRD complex and partial displacement of constituent proteins from the nucleus to the cytoplasm. Disruption of NuRD corepression correlates with accumulation of *L1* mRNA and proteins and tumorigenesis.

## Author contributions

KSR conceived the study, KSR and PBJr designed the study, PBJr acquired the data, KSR and PBJr analyzed and interpreted the data, and KSR and PBJr wrote the manuscript.

## Supporting information


**Fig. S1.** Optimization of L1‐ORF1 in HBECs by Western analysis.
**Fig. S2.** Time‐dependent analysis of *L1* mRNA accumulation in HBECs challenged with 0.1 μm BaP.
**Fig. S3.** DNA and Sequence analysis of L1‐5′UTR sequences.
**Fig. S4.** Analysis of CYP1A1 expression after 12 h of BaP treatment by indirect immunofluorescence.

## References

[mol212329-bib-0001] Allen HF , Wade PA and Kutateladze TG (2013) The NuRD architecture. Cell Mol Life Sci 70, 3513–3524. 23340908 10.1007/s00018-012-1256-2 PMC3652912

[mol212329-bib-0002] Baubec T , Ivanek R , Lienert F and Schubeler D (2013) Methylation‐dependent and ‐independent genomic targeting principles of the MBD protein family. Cell 153, 480–492. 23582333 10.1016/j.cell.2013.03.011

[mol212329-bib-0003] Boissinot S , Chevret P and Furano AV (2000) L1 (LINE‐1) retrotransposon evolution and amplification in recent human history. Mol Biol Evol 6, 915–928. 10.1093/oxfordjournals.molbev.a026372 10833198

[mol212329-bib-0004] Bojang P Jr , Anderton MJ , Roberts RA and Ramos KS (2014) De novo LINE‐1 retrotransposition in HepG2 cells preferentially targets gene poor regions of chromosome 13. Genomics 104, 96–104. 25043885 10.1016/j.ygeno.2014.07.001 PMC4157570

[mol212329-bib-0005] Bojang P Jr , Roberts RA , Anderton MJ and Ramos KS (2013) Reprogramming of the HepG2 genome by long interspersed nuclear element‐1. Mol Oncol 7, 812–825. 23648019 10.1016/j.molonc.2013.04.003 PMC5528423

[mol212329-bib-0006] Brouha B , Schustak J , Badge RM , Lutz‐Prigge S , Farley AH , Moran JV and Kazazian HH Jr (2003) Hot L1s account for the bulk of retrotransposition in the human population. Proc Natl Acad Sci USA 100, 5280–5285. 12682288 10.1073/pnas.0831042100 PMC154336

[mol212329-bib-0007] Butel JS and Rapp F (1966) Replication in simian cells of defective viruses in an SV40‐adenovirus “Hybrid” population. J Bacteriol 91, 278–284. 4285535 10.1128/jb.91.1.278-284.1966 PMC315946

[mol212329-bib-0008] Chandra S , Shiffman S , Scharf DM , Dang Q and Shadel WG (2007) Daily smoking patterns, their determinants, and implications for quitting. Smok Stat‐ Exp Clin Psychopharmacol 15, 67–80. 10.1037/1064-1297.15.1.67 17295586

[mol212329-bib-0009] Chang X , Fan Y , Karyala S , Schwemberger S , Tomlinson CR , Sartor MA and Puga A (2007) Ligand‐independent regulation of transforming growth factor beta1 expression and cell cycle progression by the aryl hydrocarbon receptor. Mol Cell Biol 27, 6127–6139. 17606626 10.1128/MCB.00323-07 PMC1952156

[mol212329-bib-0010] Denslow SA and Wade PA (2007) The human Mi‐2/NuRD complex and gene regulation. Oncogene 26, 543–5438. 17694084 10.1038/sj.onc.1210611

[mol212329-bib-0011] Dombroski BA , Mathias SL , Nanthakumar E , Scott AF and Kazazian HH Jr (1991) Isolation of an active human transposable element. Science 254, 1805–1808. 1662412 10.1126/science.1662412

[mol212329-bib-0012] Esnault C , Millet J , Schwartz O and Heidmann T (2006) Dual inhibitory effects of APOBEC family proteins on retrotransposition of mammalian endogenous retroviruses. Nucleic Acids Res 34, 1522–1531. 16537839 10.1093/nar/gkl054 PMC1401513

[mol212329-bib-0013] Feng Q , Moran JV , Kazazian HH Jr and Boeke JD (1996) Human L1 retrotransposon encodes a conserved endonuclease required for retrotransposition. Cell 87, 905–916. 8945517 10.1016/s0092-8674(00)81997-2

[mol212329-bib-0014] Gialitakis M , Tolaini M , Li Y , Pardo M , Yu L , Toribio A , Choudhary JS , Niakan K , Papayannopoulos V and Stockinger B (2017) Activation of the aryl hydrocarbon receptor interferes with early embryonic development. Stem Cell Reports 9, 1377–1386. 29107595 10.1016/j.stemcr.2017.09.025 PMC5831049

[mol212329-bib-0015] Giresi PG , Kim J , McDaniell RM , Iyer VR and Lieb JD (2007) FAIRE (Formaldehyde‐Assisted Isolation of Regulatory Elements) isolates active regulatory elements from human chromatin. Genome Res 17, 877–885. 17179217 10.1101/gr.5533506 PMC1891346

[mol212329-bib-0016] Grandi FC , Rosser JM , Newkirk SJ , Yin J , Jiang X , Xing Z , Whitmore L , Bashir S , Ivics Z , Izsvak Z *et al* (2015) Retrotransposition creates sloping shores: a graded influence of hypomethylated CpG islands on flanking CpG sites. Genome Res 25, 1135–1146. 25995269 10.1101/gr.185132.114 PMC4509998

[mol212329-bib-0017] Hattori M , Kuhara S , Takenaka O and Sakaki Y (1986) L1 family of repetitive DNA sequences in primates may be derived from a sequence encoding a reverse transcriptase‐related protein. Nature 321, 625–628. 2423883 10.1038/321625a0

[mol212329-bib-0018] Hohjoh H and Singer MF (1996) Cytoplasmic ribonucleoprotein complexes containing human LINE‐1 protein and RNA. EMBO 15, 630–639. PMC449981 8599946

[mol212329-bib-0019] Humphrey GW , Wang Y , Russanova VR , Hirai T , Qin J , Nakatani Y and Howard BH (2001) Stable histone deacetylase complexes distinguished by the presence of SANT domain proteins CoREST/kiaa0071 and Mta‐L1. J Biol Chem 276, 6817–6824. 11102443 10.1074/jbc.M007372200

[mol212329-bib-0020] Ikeda K , Shiraishi K , Eguchi A , Shibata H , Yoshimoto K , Mori T , Baba Y , Baba H and Suzuki M (2013) Long interspersed nucleotide element 1 hypomethylation is associated with poor prognosis of lung adenocarcinoma. Annals Thoracic Surg 96, 1790–1794. 10.1016/j.athoracsur.2013.06.035 23998411

[mol212329-bib-0021] Jiang CL , Jin SG and Pfeifer GP (2004) MBD3L1 is a transcriptional repressor that interacts with methyl‐CpG‐binding protein 2 (MBD2) and components of the NuRD complex. J Biol Chem 279, 52456–52464. 15456747 10.1074/jbc.M409149200

[mol212329-bib-0022] Kaur E , Gupta S and Dutt S (2007) Clinical implications of MTA proteins in human cancer. Cancer Metastasis Rev 33, 1017–1024. 10.1007/s10555-014-9527-z 25374266

[mol212329-bib-0023] Kazazian HH Jr , Wong C , Youssoufian H , Scott AF , Phillips DG and Antonarakis SE (1988) Haemophilia A resulting from de novo insertion of L1 sequences represents a novel mechanism for mutation in man. Nature 332, 164–166. 2831458 10.1038/332164a0

[mol212329-bib-0024] Lai AY and Wade PA (2011) Cancer biology and NuRD: a multifaceted chromatin remodelling complex. Nat Rev Cancer 11, 588–596. 21734722 10.1038/nrc3091 PMC4157524

[mol212329-bib-0025] Lechner MS , Begg GE , Speicher DW and Rauscher FJ 3rd (2000) Molecular determinants for targeting heterochromatin protein 1‐mediated gene silencing: direct chromoshadow domain‐KAP‐1 corepressor interaction is essential. Mol Cell Biol 20, 6449–6465. 10938122 10.1128/mcb.20.17.6449-6465.2000 PMC86120

[mol212329-bib-0026] Lu KP and Ramos KS (2003) Redox regulation of a novel L1Md‐A2 retrotransposon in vascular smooth muscle cells. J Biol Chem 278, 28201–28209. 12714586 10.1074/jbc.M303888200

[mol212329-bib-0027] Mansfield RE , Musselman CA , Kwan AH , Oliver SS , Garske AL , Davrazou F , Denu JM , Kutateladze TG , Mackay JP (2011) Plant homeodomain (PHD) fingers of CHD4 are histone H3‐binding modules with preference for unmodified H3K4 and methylated H3K9. J Biol Chem 286, 11779–11791. 21278251 10.1074/jbc.M110.208207 PMC3064229

[mol212329-bib-0028] McClintock B (1984) Significance of responses of the genome to challenge. Science 226, 792–801. 15739260 10.1126/science.15739260

[mol212329-bib-0029] Mohd‐Sarip A , Teeuwssen M , Bot AG , De Herdt MJ , Willems SM , Baatenburg de Jong RJ , Looijenga LHJ , Zatrenu D , Bexstarosti K , Van Riet J *et al* (2017) DOC1‐dependent recruitment of NURD reveals antagonism with SWI/SNF during epithelial‐mesenchymal transition in oral cancer cells. Cell Rep 20, 61–75. 28683324 10.1016/j.celrep.2017.06.020

[mol212329-bib-0030] Montoya‐Durango DE , Liu Y , Teneng I , Kalbfleisch T , Lacy ME , Steffen MC and Ramos KS (2009) Epigenetic control of mammalian LINE‐1 retrotransposon by retinoblastoma proteins. Mutat Res 665, 20–28. 19427507 10.1016/j.mrfmmm.2009.02.011 PMC3418809

[mol212329-bib-0031] Montoya‐Durango DE , Ramos KA , Bojang P Jr , Ruiz L , Ramos IN and Ramos KS (2016) LINE‐1 silencing by retinoblastoma proteins is effected through the nucleosomal and remodeling deacetylase multiprotein complex. BMC Cancer 16, 38. 26810492 10.1186/s12885-016-2068-9 PMC4727354

[mol212329-bib-0032] Moran JV , Holmes SE , Naas TP , DeBerardinis RJ , Boeke JD and Kazazian HH Jr (1996) High frequency retrotransposition in cultured mammalian cells. Cell 87, 917–927. 8945518 10.1016/s0092-8674(00)81998-4

[mol212329-bib-0033] Nanez A , Ramos IN and Ramos KS (2011) A mutant Ahr allele protects the embryonic kidney from hydrocarbon‐induced deficits in fetal programming. Environ Health Perspect 119, 1745–1753. 21803694 10.1289/ehp.1103692 PMC3261986

[mol212329-bib-0034] Pan JJ , Chang QS , Wang X , Son YO , Liu J , Zhang Z , Bi YY and Shi X (2011) Activation of Akt/GSK3β and Akt/Bcl‐2 signaling pathways in nickel‐transformed BEAS‐2B cells. Int J Oncol 39, 1285–1294. 21837363 10.3892/ijo.2011.1157

[mol212329-bib-0035] Ramírez J , Dege C , Kutateladze TG and Hagman J (2012) MBD2 and multiple domains of CHD4 are required for transcriptional repression by Mi‐2/NuRD complexes. Mol Cell Biol 32, 5078–5088. 23071088 10.1128/MCB.00819-12 PMC3510529

[mol212329-bib-0036] Ramos KS , Montoya‐Durango DE , Teneng I , Nanez A , Stribinskis V (2011) Epigenetic control of embryonic renal cell differentiation by L1 retrotransposon. Birth Defects Res A Clin Mol Teratol 91:693–702. 21384534 10.1002/bdra.20786 PMC3180906

[mol212329-bib-0037] Reyes‐Reyes E , Aispuro I , Tavera‐Garcia M , Field M , Moore S , Ramos I and Ramos KS (2017) LINE‐1 couples EMT programming with acquisition of oncogenic phenotypes in human bronchial epithelial cells. Oncotarget 8, 103828–103842. 29262603 10.18632/oncotarget.21953 PMC5732769

[mol212329-bib-0038] Reyes‐Reyes EM , Ramos IN , Tavera‐Garcia MA and Ramos KS (2016) The aryl hydrocarbon receptor agonist benzo(a)pyrene reactivates LINE‐1 in HepG2 cells through canonical TGF‐β1 signaling: implications in hepatocellular carcinogenesis. Am J Cancer Res 6, 1066–1077. 27293999 PMC4889720

[mol212329-bib-0039] Saito K , Kawakami K , Matsumoto I , Oda M , Watanabe G and Minamoto T (2010) Long interspersed nuclear element 1 hypomethylation is a marker of poor prognosis in stage IA non‐small cell lung cancer. Cancer Res 16, 2418–2426. 10.1158/1078-0432.CCR-09-2819 20371677

[mol212329-bib-0040] Scott AF , Schmeckpeper BJ , Abdelrazik M , Comey CT , O'Hara B , Rossiter JP , Cooley T , Heath P , Smith KD and Margolet L (1987) Origin of the human L1 elements: proposed progenitor genes deduced from a consensus DNA sequence. Genomics 1, 113–125. 3692483 10.1016/0888-7543(87)90003-6 PMC7135745

[mol212329-bib-0041] Stribinskis V and Ramos KS (2006) Activation of human long interspersed nuclear element 1 retrotransposition by benzo(a)pyrene, an ubiquitous environmental carcinogen. Can Res 66, 2616–2620. 10.1158/0008-5472.CAN-05-3478 16510580

[mol212329-bib-0042] Swergold GD (1990) Identification, characterization, and cell specificity of a human LINE‐1 promoter. Mol Cell Biol 10, 6718–6729. 1701022 10.1128/mcb.10.12.6718-6729.1990 PMC362950

[mol212329-bib-0043] Takeshita K , Ogawa HI and Maeda T (2016) Structural chromosome aberrations cause swelling of the nucleus. Genes Environ 38, 22. 27733886 10.1186/s41021-016-0047-7 PMC5045629

[mol212329-bib-0044] Teneng I , Montoya‐Durango DE , Quertermous JL , Lacy ME and Ramos KS (2011) Reactivation of L1 retrotransposon by benzo(a)pyrene involves complex genetic and epigenetic regulation. Epigenetics 6, 355–367. 21150308 10.4161/epi.6.3.14282 PMC3092684

[mol212329-bib-0045] Teneng I , Stribinskis V and Ramos KS (2007) Context‐specific regulation of LINE‐1. Genes Cells 12, 1101–1110. 17903170 10.1111/j.1365-2443.2007.01117.x

[mol212329-bib-0046] Wang Y1 , Zhang H , Chen Y , Sun Y , Yang F , Yu W , Liang J , Sun L , Yang X , Shi L *et al* (2009) LSD1 is a subunit of the NuRD complex and targets the metastasis programs in breast cancer. Cell 138, 660–672. 19703393 10.1016/j.cell.2009.05.050

[mol212329-bib-0047] Wei W , Gilbert N , Ooi SL , Lawler JF , Ostertag EM , Kazazian HH , Boeke JD and Moran JV (2001) Human L1 retrotransposition: cis preference versus trans complementation. Mol Cell Biol 21, 1429–1439. 11158327 10.1128/MCB.21.4.1429-1439.2001 PMC99594

[mol212329-bib-0048] Wylie A , Jones AE , D'Brot A , Lu WJ , Kurtz P , Moran JV , Rakheja D , Chen KS , Hammer RE , Comerford SA , *et al* (2016) p53 genes function to restrain mobile elements. Genes Dev 30, 64–77. 26701264 10.1101/gad.266098.115 PMC4701979

[mol212329-bib-0049] Xue Y , Wong J , Moreno GT , Young MK , Cote J and Wang W (1998) NURD, a novel complex with both ATP‐dependent chromatin‐remodeling and histone deacetylase activities. Mol Cell 2, 851–861. 9885572 10.1016/s1097-2765(00)80299-3

[mol212329-bib-0050] Zhang Y , Ng HH , Erdjument‐Bromage H , Tempst P , Bird A and Reinberg D (1999) Analysis of the NuRD subunits reveals a histone deacetylase core complex and a connection with DNA methylation. Genes Dev 13, 1924–1935. 10444591 10.1101/gad.13.15.1924 PMC316920

