# Supplementary material for: Epigenetic reactivation of LINE‐1 retrotransposon disrupts NuRD corepressor functions and induces oncogenic transformation in human bronchial epithelial cells
Source: Mol Oncol. 2018 Jun 21;12(8):1342–57. doi: 10.1002/1878-0261.12329 (PMC6068357; doi:10.1002/1878-0261.12329)
Supplement: Supplementary file 1 — Fig. S1. Optimization of L1‐ORF1 in HBECs by Western analysis. Fig. S2. Time‐dependent analysis of L1 mRNA accumulation in HBECs challenged with 0.1 μm BaP. Fig. S3. DNA and Sequence analysis of L1‐5′UTR sequences. Fig. S4. Analysis of CYP1A1 expression after 12 h of BaP treatment by indirect immunofluorescence. [file MOL2-12-1342-s001.pdf]

Bojang and Ramos Supplementary Figure 1:

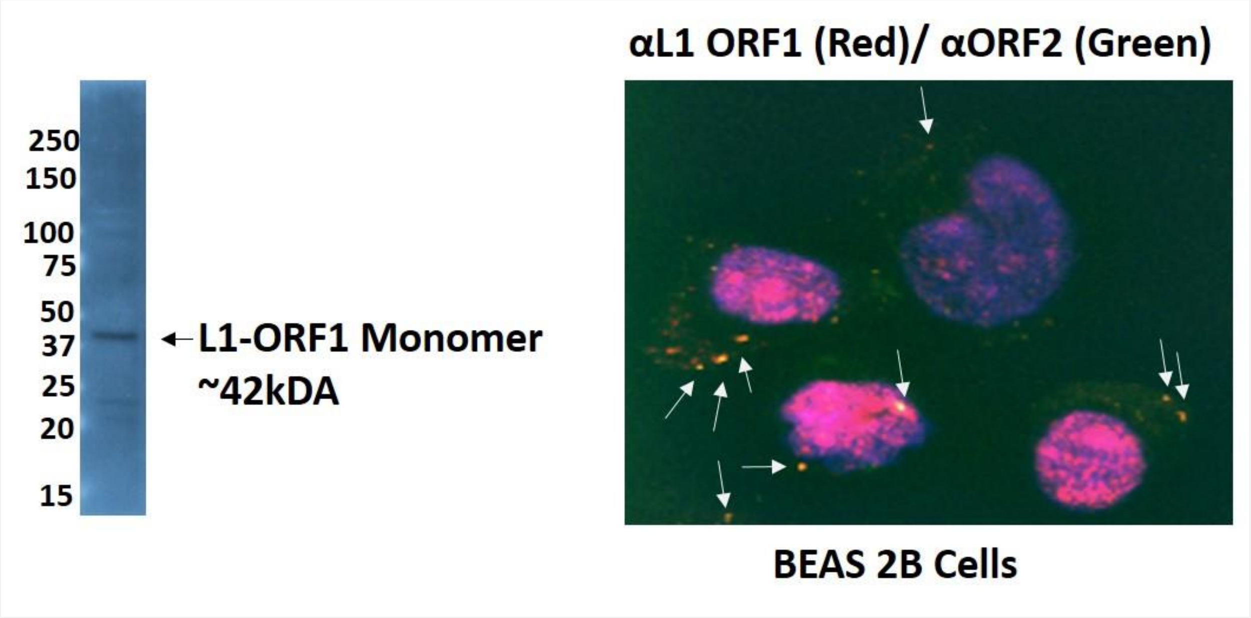

Supplementary Figure 1: Optimization of L1-ORF1 antibody in HBECs.

Bojang and Ramos Supplementary Figure 2:

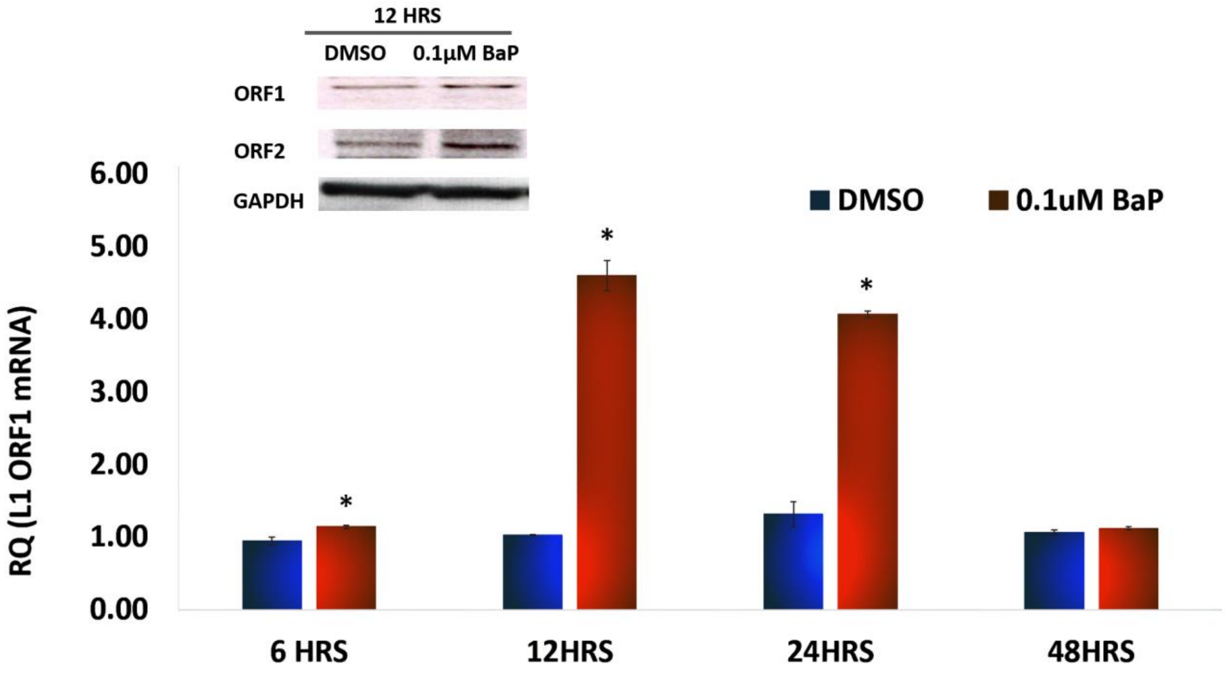

Supplementary Figure 2: Time dependent analysis of *L1* mRNA accumulation in HBECs challenged with 0.1 μM BaP. RT-PCR analysis of L1 expression in HBECs challenged with DMSO vehicle or 0.1μM BaP for 6, 12, 24, or 48 hr. Equal volumes of DMSO were added to all plates. Western analysis confirming the induction of L1 proteins at the 12 h time point. Significant difference was determined by Mann-Whitney U Test.

Bojang and Ramos Supplementary Figure 3:

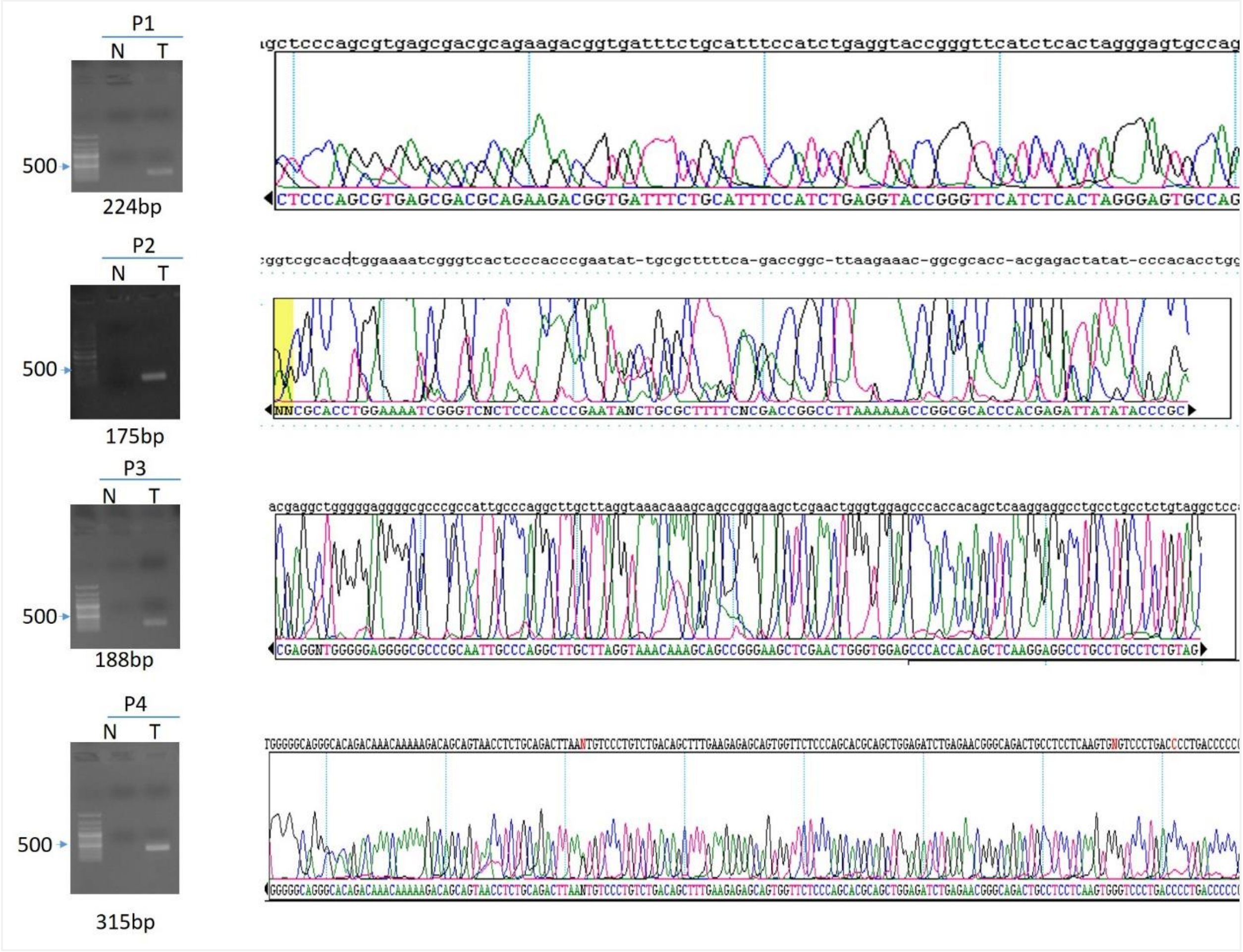

Supplementary Figure 3: DNA and Sequence analysis of L1 5'UTR sequences. Enriched sequences are of the expected sizes (P1: 224bp, P2: 175bp, P3: 188bp and P4: 315bp) and are identical to L1 5'UTR DNA sequence.

Bojang and Ramos Supplementary Figure 4:

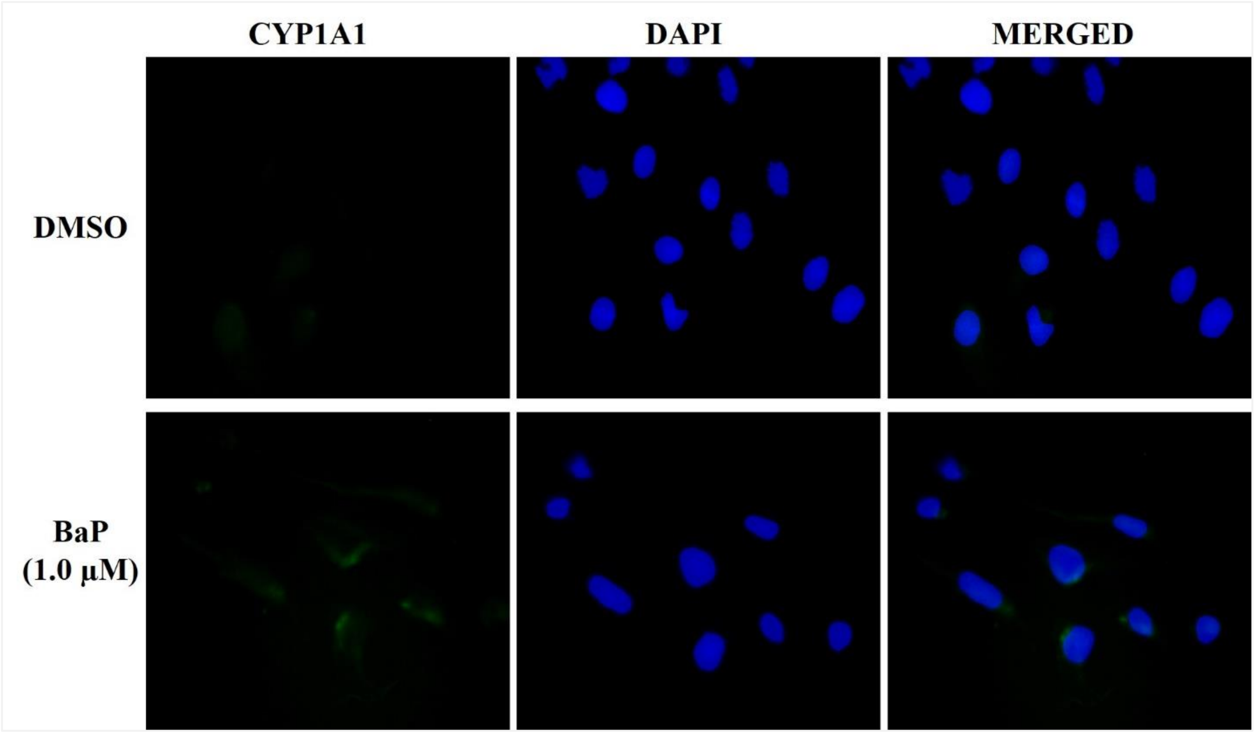

Supplementary Figure 4: Analysis of CYP1A1 expression after 12 h of BaP treatment by indirect immunofluorescence.
